# A benchmarking program to support software process improvement adaptation in a developing country, a Pakistan case

**DOI:** 10.7717/peerj-cs.936

**Published:** 2022-04-27

**Authors:** Umema Hani, Khalid Khan, Usman Amjad, Noor Zaman Jhanjhi, Ayub Latif, Saood Zia

**Affiliations:** 1 COCIS, Karachi Institute of Economics and Technology, Karachi, Sindh, Pakistan; 2 School of Computer Science & Engineering, Taylor’s University, Subang Jaya, Selangor, Malaysia; 3 Software Engineering, Sir Syed University of Engineering and Technology, Karachi, Sindh, Pakistan

**Keywords:** Productivity improvement, Performance improvement benchmarks, Software process improvement, CMMI benchmarks, Quality management

## Abstract

**Background:**

Standardization of software development processes using the software process improvement (SPI) frameworks like capability maturity model integration (CMMI) or the International Organization for Standardization (ISO) are vital for better performance and defect-free delivery of software engineering projects.

**Problem:**

The studies from developing countries show that the organizations are unable to get the desired SPI benefits during early adaptation due to lack of benchmarking initiatives. These initiative needed bridging of the gap between major software engineering fields such as project performance, productivity, and SPI measurement metrics like effort, schedule, cost, productivity, quality, and customer satisfaction.

**Method:**

This research quantifies SPI benefits by bridging the existing gaps and identifying a commonly defined set of 44 significant base metrics related to ESCPQC and applies a conceptual framework over the collected data of 62 projects from three leading software organizations.

**Result:**

The results have quantified the “the introduction of change effect” and showed that the adaptation of SPI has shown SPI benefits like improved quality, customer satisfaction, and effort. In contrast, schedule metrics showed a high reduction at level 3 and again increased at high maturity levels values. The last benefits are communicated following a trustable mechanism.

## Introduction

There is a common perception in general that “following a standard could curtail one’s creativity and hinder the progress of their work”. Moreover, following organizational capability or maturity standards reduces the activity of reinventing the same procedures and having good grip on the results in a reliable software product (Jarvis & Crandall, 1997).

Before investing in software process improvement (SPI) initiatives, there is a strong need for SPI benefit quantification to gain its clear understanding as communicated in SPI studies conducted for different countries like Spain, Canada, Pakistan, Indonesia, and Nepal, etc. (Khurshid, Bannerman & Staples, 2009; Chevers & Duggan, 2010; Garzás & Paulk, 2013; Ahmed et al., 2016; Alqadri et al., 2020), among which Bangladeshi software firms have not experienced much in this particular area in comparison to other countries, In context to Nepal, only two software companies are capability maturity model integration (CMMI) appraised (Thapa, 2016). Whereas, in Pakistan only one company has attained CMMI level 5 (Ahmed et al., 2016). The perception of the inconclusive benefits of SPI programs affects the decision of many companies to implement such programs, which is also one of the most occurring factors discussed in Critical Failure Factor (CFF) that causes hindrance in SPI adaptation (Ahmed et al., 2016). The benefit quantification will pave the way for establishing a reliable cross-organizational benchmark effort, where benchmarking is the practice of comparing business process wise performance metrics to industry best practices from other companies.

The Pakistan Software Export Board (PSEB) of Pakistan has already assisted 110 companies in achieving ISO 9001, 11 companies in ISO27001, 23 companies on CMMI level 2, Level 3 & Level 5. PSEB is now looking forward to assist 30 more companies at various level of CMMI and 50 companies on ISO 27001 & ISO 20000 in the next 5 years. The policy shows the popularity of ISO 9000:2001 and CMMI process standardization in Pakistan as given on a web source (Pakistan Software Export Board (PSEB), 2008). Recently CMMI version-2 has been released (ISACA, 2020) by ISACA and its benefits are also shared publically. There is a need to develop a deeper understanding of CMMI version 2 and its impact on process improvement as it is more focused on productivity and going hand in hand with Agility and DevOps for around 50% participant companies where as the rest of the organizations must rely on other evolutionary process model like waterfall, incremental, RUP and other software development life cycle models. Therefore, for the beginners there is a need to get a true picture of core SPI behavior independently (ISACA, 2020).

### Problems and research motivation

Different study reports reveal many failure rates in SPI adoption and their possible reasons. The ones targeted in this study are given bellow:

- The unavailability of the similar scale project’s SPI benefit data (Khurshid, Bannerman & Staples, 2009).
- The emergency of reaching towards the expected results to get early certification and the use of manual methods of data collection causes biases in historically collected metrics data and the creation of gold platted evidences for SPI appraisals (Garzás & Paulk, 2013).
- The shortage of benchmarks or empirical data on SPI implementation (Hassan et al., 2014).

However, in spite of having some relevant studies like Ii & Goldenson (2010) and Jones (2014) that has identified the details on ESCPQC gains in percent increase or decrease.

- There is inconsistency in terms of common metric definitions across different organizations that results into inconsistent categorization of results, as the inconsistent information is impossible to get statistically verifiable, benchmarking is impossible McLoughlin & Richardson (2010) and Kasunic (2008).

In current benefit studies ISACA (2020), Ii & Goldenson (2010), Alqadri et al. (2020), McLoughlin & Richardson (2010), the data has emerged for the technologically advanced countries, and does not directly addresses the influencing factors of SPI adaptation for the developing countries thus left its effects debatable. Following the recommendation highlighted in a study (Salleh, Bahari & Ismail, 2020), which is especially conducted for economically developed country Pakistan, emphasis that the organization structure and political instability had a significant difference across developed, developing and semi-industrialized countries when compared country-wise.

- The study encourages researchers to analyze the future empirical studies based on a specific region to validate the suitability of these factors in a specific country (Salleh, Bahari & Ismail, 2020).

Since, the isolated and varying approaches of reporting SPI benefits will neither let us effectively share our data nor could the organizations participate in establishing cross-organizational benchmarks to facilitate the quantification of SPI benefits. Therefore, this research raises the need for developing a persuasive benchmarking program for SPI benefits justification for its adoption.

### Proposed research scheme

**Figure 1:**
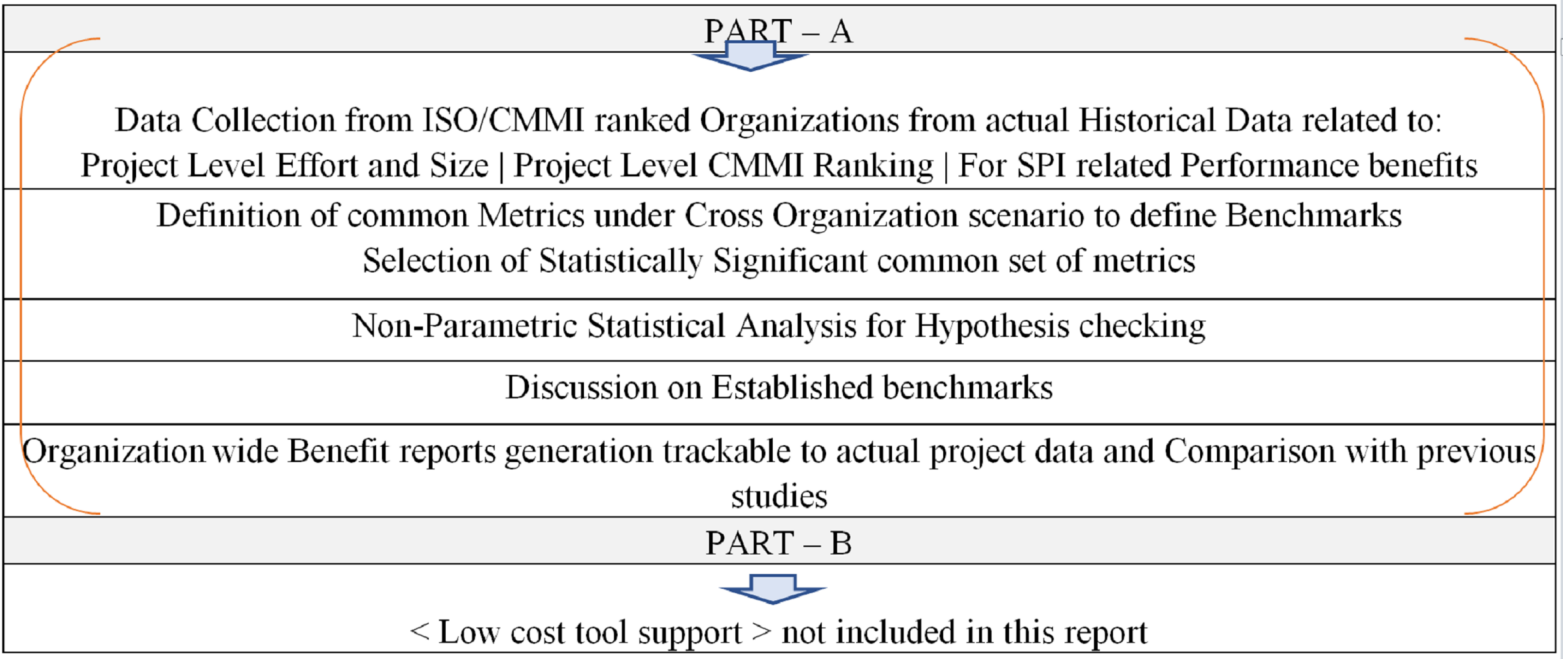
Research scheme.

Figure 1 presents the overall research scheme. Part A is about data collection from CMMI appraised organizations and the selection of statistically significant metrics under cross-organization scenario to define benchmarks. The “Benefits Reports” reflects the achieved benefits of adapting different SPI levels, reflected in the form of SPI benefit measurement classes ESCPQC.

These reports are built upon the project level prediction data, which may then be generalized in percent form to be presented for assessment during CMMI appraisals designated as Class C, which are conducted in-house by the Process Management team of the respective organization. Part-B of this research is about the enablement of predictability for ESCP classes on a small dataset, which is a part of Future work.

### Purpose and novelty of research

The purpose of this study is to achieve the following goals:

- To statistically examine and quantify the relationship and its strength between SDPI levels and benefit measurement classes namely effort, schedule, cost, productivity, quality, and customer satisfaction (ESCPQC).
- To develop base level metrics or factors picked up from different software engineering fields that will make the pave of cross organizational performance benchmarks.
- To report benefit benchmarks and benefits reports.
- This study is initially intended for the software development organizations of developing countries like Portugal, Indonesia, and Pakistan to invest more effectively in SPI programs instead of wasting on certification that has little or no impact.

As far as research novelty is concerned, to our knowledge, the scheme of generating benefits directly from organizational project data and formation of cross-organizational benchmarks to predict the situation of quality attributes, productivity parameters, or the major benefit measurement classes ESCPQC in the context of SPI process performance measurement, are novel features of our approach.

## Related Work and Gap Analysis

This section covers a detailed literature review on the major problems that were identified in literature available on the quantification of SPI benefits and its constituent areas.

During the literature review we searched all the possible articles on SPI benefit measurement studies using the following phrases on relevant repositories Web of Science, ACM, IEEE, and Springer:

1. CMMI and ISO based software process improvement.
2. CMMI and ISO based software process improvement benefits.
3. CMMI and ISO based software process improvement performance benefits.
4. Critical success factors in ISO and CMMI.
5. Impact of productivity parameters on software process improvement.
6. Impact of software process improvement on effort, schedule, and cost.

Then the searched articles were reviewed for relevance in this study and their referenced articles were further explored to track the information depth. It is suggested in Kasunic (2008), Khurshid, Bannerman & Staples (2009), McLoughlin & Richardson (2010), the SPI benefits can be fully achieved through benchmarking initiatives.

This research covers multiple deficiencies related to these pending benchmarking initiatives by utilizing three important areas of software *i.e.*, “Performance Improvement Benchmarking”, “Process Standard Frameworks”, and “Software Development Prediction Models” also known as metrics and productivity parameters. The gap between these areas is reduced to generate the SPI benchmarks by linking and utilizing the areas’ required parameters, as shown in Fig. S1. It shows that all three areas has some identified problems addressed in this research for SPI benefits benchmarking and report generation in a cross organization environment. The targeted problems are already discussed in detail in “Problems and Research Motivation”.

The research done in “Software Development Prediction Models” is utilized to select different measurement metrics or productivity parameters to measure the SPI benefits. Major metric definitions are picked from the Constructive Cost Model (COCOMO) model definition manual for effort, schedule, cost, and productivity classes. There is series of researches to make COCOMO more efficient using different algorithms and techniques for higher accuracy in estimating software effort (Ghatasheh et al., 2015; Wu & Kuan, 2017; Kaur et al., 2018; Wani, Giri & Bashir, 2019; Nassif et al., 2019; Nguyen, Boehm & Huang, 2019; Priya Varshini & Varadarajan, 2021).

In the CMMI based “Performance Improvement Benchmarking” area a technical report is recently published by CMMI Institute that shares performance report summary for projects appraised in year 2019 using the latest version of CMMI (ISACA, 2020). The latest contribution in this area attempts to facilitate the process’s performance analysis with the help of a tool kit to automate the “Performance Measurement Analysis” to reduce the difficultly faced in a manual process because of its dependency on benchmarking of data (Raza & Faria, 2020). However, the understanding for using the kit is time-consuming that increases the “schedule factor”. Another study presents the CMMI based SPI benefit benchmarks of level 5 projects and showed the average benefits in terms of software development productivity up to 50% (Pai, Subramanian & Pendharkar, 2015).

In the “Process Standard Frameworks” area a list of references are found that highlights the Critical Failure Factors (CFF) that are important for the success of SPI implementation or otherwise creates hurdles. A study conducted for developing countries, from thirty three software companies of Pakistan who have implemented CMMI. A total of 48 software practitioners and 4 SPI professionals who have participated in this research revealed that time and budget constraints and cultural issues are the most impacting ones. Whereas, there have been responses among respondents with different job functions as the top management mostly disagrees with all issues. Surprisingly, most of the respondents with middle and lower management agree with the existence of these issues in the IT Industry (Ahmed et al., 2016). Another study finds out CFF for implementing CMMI-DEV against the problems faced in Indonesia’s implementation. The identified hindrance factors includes lack in experienced SPI staff, dissatisfaction of staff and high turnover, politics as a result of reallocation of resources, lack of higher management support in financing, realizing, and implementing SPI, low budget allocation and sponsorship for SPI project, cultural language issues and trust building issues, personality clashes, consideration of practitioner’s feedback before next implementation, communication barriers in between the practitioners and the assessors, understanding and implementation of formal methodology followed, add-on workload with regular tasks, time pressures that affects decision, company infrastructure, distance between the assessors and practitioners, and Implementation tools and standards show indicators of tools used in SPI implementation and experienced staff (Alqadri et al., 2020).

Khan et al. (2019) identified CFF factors that hinder SPI performance and frequency of occurrence in survey. The study suggests the most occurring failure factor as lack of understanding of SPI with frequency of 80% and budget shortage with frequency 85%. Due to above mentioned constraints it becomes difficult to hire SPI consultants (Khan et al., 2019). Whereas, Niazi, Babar & Verner (2010) in a similar study has highlighted two other CFF that might take over SPI failure, one is “need to adopt proper tools for SPI implementation” and second is “motivate team members to have sufficient SPI tool knowledge”.

The findings of another research for small and medium sized enterprises conclude that organization, people, and technology issues are important factors that need to be focused on for a successful implementation of SPI (Sharma & Sangal, 2019).

From above screening of the latest relevant research undergone in all the three areas as mentioned in Fig. S1, it can be observed that Literature has lost the focus from the benchmarking of “SPI performance benefits”. The major studies that have discussed on the identification of SPI implementation level CFF for the successful delivery of quality frameworks in developing countries such as Indonesia (Alqadri et al., 2020), Pakistan (Shah et al., 2012), and (Hassan et al., 2014) point outs the need of benchmarking to understand the benefits of SPI quantitatively. Still there is no work done for the quantification of SPI benefits, and SPI benefit data is available in the form of case studies, practitioner testimonials, press releases, and public relations. Even an attempt has been made to bypass the manual process of “Performance Analysis” which needs benchmarking.

Table S1 covers the gap analysis summary after going through the relevant literature review to identify the problems, find out motivation, and establish a base to come up with a framework for “Effective SPI benefits’ Reporting & Cross-Organizational Benchmarks”. See “Problems and Research Motivation” for an overview of problem identification and research motivation. One relevant case study (Ii & Goldenson, 2010) discusses CMMI based performance results across ten organizations and uses “variant metrics definitions” across different organizations for the same benefit class. For example, the varying metric definitions used for measuring schedule includes “Variation in schedule”, “Number of days late”, “Days variance from the plan” and “Schedule performance index”. And the latest benefit benchmarks are released in study (ISACA, 2020).

A drawback of using this variant approach is that, to know the benefits of SPI in advance, the interested organization has to search in the individual case study and check its applicability on his business scenario. The available studies are not comparable and generalized across different organizations.

The research (McLoughlin & Richardson, 2010) highlights the major hurdles in establishing a smooth benchmarking program for SPI, *i.e.*, lack of standardization and normalization. It suggests the possibility of making benefit benchmarks on (1) reduced cost of IT operation as they believe it is comparable across the same industry and their cultures. (2) Increased productivity with regard to function point is comparable, and level of quality could be benchmarked easily after standardization where reduced cycle time (depends on type of processes, tools, and targeted product make it difficult to compare) and increased revenue are difficult to get normalized (depending upon market and product type and could be generalized within the domain). Such inconsistencies are obvious as they speak about the actual results of SPI initiatives reported from different organizations, of different size in different industries with different constraints and independently carried out. Although, Hassan et al. (2014) proposed a compensating statistical procedures and an additional factor “infrastructure effort points” is presented, which could be used so that different project data can be compared in a meaningful way based on similar project characteristics. However, this study has targeted data collection from multiple successful CMMI, and ISO appraised organizations involved in software development and identification of basic metric set. The first challenge of this research was to come up with a kind of metrics package that could be used to identify a basic set of metrics that comes under the category of SPI “benefit measurement classes” such as ESCPQC. After an extensive literature review a detailed set of relevant metrics were identified. Let us go through some most relevant references from existing studies that have been used to build upon a strong base for making a list of common metric definitions usable across cross organizations.

Statz (2005) proposed complete guidance is given by the Practical Software and System Measurement (PSM) community of the US department of defense for software and system projects on the measurement of process improvement. It covers the measurable concepts of information and their mapping with perspective measures used by the projects. Majority of the metrics in this study are taken from this guidance study. Another research (Unterkalmsteiner et al., 2012) has been considered while selecting ESCPQC related metrics, it identifies frequencies of usage for benefit measurement metrics or success indicators in different organizations. In the study of Rico & Keagle (1999) discusses different classes of metrics used by different researchers and organizations like Software Engineering Institute (SEI), Data and Analysis Center for Software (DACS) and Software Productivity Research (SPR) as a source of SPI cost and benefits. These metrics comprises of defects, cost invested, precision, costing, sizing, years, and people. Jones & Bonsignour (2011) discusses the reliability issue of lines of code (LOC) and empirically proves it as a bad way to measure software development output under all software development conditions. It debates that it is false if what is consistently followed is bad practice. But, unfortunately the reported Function Point data does not shows significant result when analyzed statistically (see “Experimental Design and Results”). Another observation found in Ii & Goldenson (2010), Gibson, Goldenson & Kost (2006), Mcgibbon, Ferens & Vienneau (2007) is that they have communicated SPI benefits in percent improvement form. For example, the Siemens information system shows the reduced defect density an average of 71% in three technical areas. The currently available data on ESCPQC gains is in the form of a percentage of improvement in related metrics and these benefit percentages are not trackable to real project level data.

This study targets a study region as economically developing country, Pakistan. It undergoes a thorough statistical significance analysis, generalization of measurements at Project Level, while being in compliant to the solutions as proposed against problem 1 and 2 by using the defined basic metric definitions for cross organization-wide scenarios, benefits reported in percent form, using project-level data in actual metric values that later on could be converted into a higher view of within an organization benefits in percentage form.

## Methodology

### Research method and design

The design of this empirical research is a quantitative, observational, and statistical study based on evidence data collection in alignment with its targeted goals as discussed in “Introduction”. The study set out to answer the specific research questions. The overall methodology is similar to study (Agrawal & Chari, 2007), see Fig. 1 for higher level research steps. Literature review is performed on targeted area and identified gaps in existing work. Literature Review includes the collection of email-based feedback from practitioners and field experts for the confirmation of the usefulness of the selected topic, confirmation of relevance of the factors identified through literature, and finding a mechanism and metric to measure benefits. After that Research Questions and Hypotheses are defined in context of relevant work done. Then sample is identified from the targeted data. Measurement instrument was selected and pilot testing is performed before its finalization. On collected data the data cleaning is performed and then detailed experimentation is performed to generate final benchmarks. While doing so, a conceptual model is designed to evaluate the relationships between SDPI levels and project performance parameters or benefit measurement classes. Data analysis and interpretation are performed, and experimental results are generated to identify significant basic set of metrics and derived conclusions regarding the behavior of benefit measurement metrics. Experimental results are generated to quantitatively generate Benefit reports and then in percent form along with derived conclusion regarding the behavior of benefits metrics used.

### Research question and hypothesis

Two research questions are being addressed in this research:

1. The first research question (RQ1) states that: is there any change observed in the medians of effort or schedule or cost or productivity or quality or customer satisfaction (ESCPQC) when their organization has attained any of the increasing SDPI levels.
2. Whereas, the second research question (RQ2) states that: quantify exactly the strength of relationship between the SPI levels and the different ESCPQC related metric value.

To investigate above research questions, a detailed set of hypotheses were made based upon studies which have studied SPI impact over varying parameters such as productivity, schedule and effort (Ii & Goldenson, 2010; Gibson, Goldenson & Kost, 2006; Mcgibbon, Ferens & Vienneau, 2007; Agrawal & Chari, 2007). Following a more understandable structured way, six hypotheses are posted representing each SPI measurement class (ESCPQC), about checking whether the increasing levels of SPI produces a change in (E, S, C, P, Q, and C), means do they have a significant relationship? Following are the posted hypothesis:

- H1: Increasing levels of SPI produce a change in E, or they have a significant relationship.
- H2: Increasing levels of SPI produce a change in S, or they have a significant relationship.
- H3: Increasing levels of SPI produce a change in Co, or they have a significant relationship.
- H4: Increasing levels of SPI produce a change in P, or they have a significant relationship.
- H5: Increasing levels of SPI produce a change in Q, or they have a significant relationship.
- H6: Increasing levels of SPI produce a change in Cs, or they have a significant relationship.

The above six hypotheses (H1 to H6) are statistically interpreted as hypotheses the increasing level of SPI are correlated to project performance parameters ESCPQC.

### Demographic information

The demographic information of respondents shows data credibility and strength. The respondents were experienced enough to understand software development practices to a level necessary to provide a qualified assessment. They were the designated Quality Managers with approximate 10 years of relevant experience and had formal CS degrees in computer science which shows that they had a good understanding of concepts such as qualitative variable or empirical estimation models. The historical project data was collected from three CMMI appraised organizations having a valid certification. All participant organizations were Class C appraised, which is considered as the most authentic appraisal level done by the SEI certified team. Whereas, post-accreditation three years is the maximum validity range for both CMMI and ISO. The collected data was compiled to analyze what different companies have experienced in implementing diverse ISO and CMMI models.

### Targeted population and sampling method

Following the data protection policy, project numbers were allocated to each participating organization which has been used throughout the data collection and validation phase instead of using the organization name. All companies were assessed by formal appraisal bodies in Pakistan and were considered well-established IT companies in Pakistan. Table 1 shows the company wise data collection pattern by mapping the maturity level wise project dataset used in the Analysis and initial dataset collected. The convention used here is as first comes the maturity level then in bracket first comes the (number of datasets actually used and the number of datasets collected). For example, 0 (15/18) means from company one at maturity level 0 a total of 18 project datasets were returned, but only 15 were selected for analysis. Projects could be also categorized according to their business domain.

**Table 1:** Company wise detail of collected project data.

Company number	Company wise project mapping: Maturity level (Number of datasets used/Number of datasets initially collected)	Project business domain areas	Life cycle model	Size	Inclusion of questionnaire and datasets in study
1	0 (15/18), 1 (10/14), 2 (6/7)	Health care	Iterative model	Medium	√
2	3 (6/7), 4 (4/4), 5 (17/17)	Finance		Large	√
3	4 (4/4)	Mortgage		Medium	√
4	2 (0/15) Incomplete fields	Apparel		Medium	X

### Data collection process

A questionnaire was designed and used the data collection process after checking its validity. The projects included in the analysis are within a size limit of a minimum 2,000 lines of code and phone interviews were used to assure high respondent motivation that results in low interview bias (Hole, 2015). The data has been collected through a questionnaire over a six-month period in this research.

### Pilot testing

Initially, pilot testing of the questionnaire was performed in which the initial questionnaire was filled from one CMMI level-2 appraised organization. During this exercise, some fields were discarded as they were not known by the organization and some fields were further disintegrated into multiple fields like the field “Defects found” was replaced by three different fields related to the defects found in (SRS, Architecture and Design, and Code).

### Data collection and response rate

Four high maturity organizations has been participated in this study and shared their historical project data. Be-cause of the missing data approximately 15 datasets were discarded from the 4th organization out of a total of 75 projects data, which make the response rate equal to 75%.

The sample finally used for the analysis comprised of 62 project data from three software development companies, comparable to the data set used in studies (Gibson, Goldenson & Kost, 2006; Agrawal & Chari, 2007; Ii & Goldenson, 2010). The mapping of the data set to the company from the 62 collected dataset is given in Table 1. The company size influences with SPI studies, usually there are two types: small and medium enterprises and large organizations. This study is not focusing over just one size of organizations and includes data from medium to large size organizations. The size definition given in Table 1 will also help in linking the organization size considered under study while targeting the “economically struggling” countries involved in outsourcing. The categorization of large and small organizations uses (a) the number of its professional and technical employees with more than 50 employees being the cut-off for large and 10–20 employees for small organizations, (b) with revenues of $200,000 (PKR 25 million) being the cutoff. All the companies are export focused local firms and have grown from zero to approximately a million dollars and employ thousands of professionals (Pakistan Software Export Board (PSEB), 2008).

SPI studies are influenced with the company size, usually there are two types: small & medium enterprises and large organizations. This study is not focusing over just one size of organizations and data from medium to large size organizations is used in this study. The size definition given in Table 1 will also help in linking the organization size considered under study while targeting the “Economically struggling” countries involved in outsourcing.

### Questionnaire design

Figure S2 shows selective parts of the actual research questionnaire for this research, which comprises different quantitative variables. The COCOMO related qualitative variables have been used in their core setting as given in (Boehm et al., 2009).

#### Variables used

Table S2 covers the definitions of major quantitative metrics used in questionnaire of this study. Majority of the definitions are taken from Statz (2005) and Boehm et al. (2009).

#### SPI scale

Before applying further data analysis on collected data there was a need for initializing a qualitative variable of “Process Maturity Rating” (PMAT) or SPI. See Table 2 for the interpretation of SPI levels of this study. These variables represent varying increasing levels of SPI. For the right assignment of increasing SPI levels *i.e.*, (1, 2,…, 5), verification was performed while matching its increasing values with the increasing ranks used in other fields.

**Table 2:** SDPI score.

SPI: SDPI levels across (ISO and CMMI) for this study	Productivity parameters: corresponding PMAT levels used	Description of SDPI levels
0	0	No SDPI framework
1	1	ISO 9001/CMMI Maturity Level 1
2	2	CMMI Maturity Level 2
3	3	CMMI Maturity Level 3
4	4	CMMI Maturity Level 4
5	5	CMMI Maturity Level 5

## Experimental Design and Results

### Conceptual model of research

**Figure 2:**
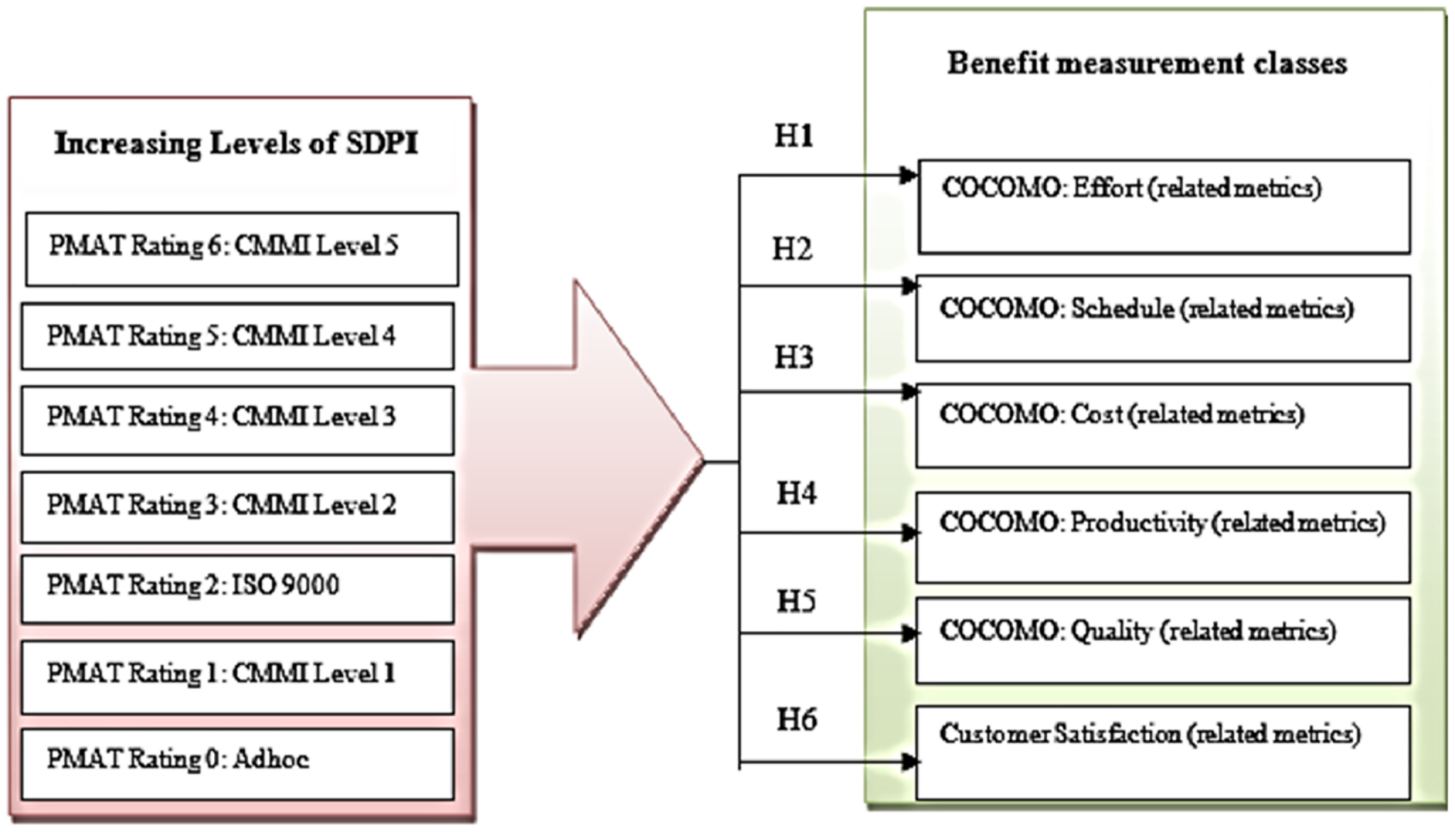
Conceptual model of SPI benefit measurement.

A conceptual model of this research identifies a set of metrics representing benefits which are common across multiple organizations. It is developed for the evaluation of the relationships between the different levels of software process improvement and project performance measurement parameters or benefit measurement classes (see Fig. 2).

The different levels of SPI includes “PMAT rating 0” represents adhoc process, “PMAT Rating 1” represents CMMI level1 process, “PMAT Rating 2” represents ISO 9000, “PMAT Rating 3” represents CMMI Level 2, “PMAT Rating 4” represents CMMI level 3, “PMAT Rating 5” represents CMMI Level 4, and “PMAT Rating 6” represents CMMI Level 5 Rating. The conceptual model consists of six hypotheses relating the levels of SPI to different benefit measurement classes, which include Effort, Schedule, Cost, Productivity, Quality, and Customer Satisfaction (see Table 2).

### Data analysis methods

As mentioned in “Methodology”, for quantitative research Statistical Package for Social Sciences (SPSS) statistical tool was used to perform analysis. The major analysis steps performed include following:

1. Research reliability to check the reliability of measured data.
2. Exploratory Data Analysis (EDA) reveals data symmetry across normal curves using measures like median and standard deviation, which is a measure of dispersion.
3. Descriptive statistics were performed to find the mean value of PMAT variable on changing levels of SPI.
4. A normality distribution test was performed using Shapiro-Wilk normality (SWN).Rank Correlation (Spearman) method was applied followed with the Non-Parametric Kruskal Wallis test to calculate the hypotheses, as both the methods support non parametric data and support data categorization according to the grouping variable *i.e.*, SPI or PMAT. From Kruskal Wallis test the effect size was also calculated to know the percent variance explained.

### Questionnaire reliability

First, the quality experts validated the questionnaire, and later its reliability was checked. Cronbach’s Alpha test was applied in order to verify the uniformity among different variables. This test was not applicable to productivity and customer satisfaction because none other field was dependent on them. For ESCPQC classes it shows values as 0.62, 0.729, 0.615, N/A, 0.803, 0.977, 1, and N/A respectively. The test results indicate that this study meets the requirements for reliability as Cronbach’s alphas ranged in between 0.6 to 1 which is under the standard acceptable reliability level (Saunders, Lewis & Thornhill, 2009).

### Exploratory data analysis

In EDA, possible errors in the data were identified and basic data trend has been observed as recommended in the study (Saunders, Lewis & Thornhill, 2009). The selection of summary measures has been done carefully depending on different data types and the level of measurement. Independent data variables used here are objective, quantitative, and continuous in nature. Whereas the dependent variable SDPI is measured as an ordinal ranking, the data values indicate both the order of values and the distance between values; the mean value was selected as a measure of central tendency. Here SPI is used to group down all independent variables.

Table S3 shows Descriptive Statistics of a sample metric variable *i.e.*, pre release defects left in code with “N” number of data points, mean, min, max and standard deviation for each changing level of SDPI, see Table 1 for its definition. Due to space restriction, only its single entry is shared in Table S3. It has been observed that data is showing different mean value across changing levels of SPI therefore, we can check the posted hypotheses either using para-metric or non-parametric methods.

### Test of normality

Before proceeding to any of these tests, data normality has been checked by applying of Shapiro-Wilk normality (SWN) tests its prerequisite first graphically using frequency histograms among which some have shown improved normal distribution on applying the natural Log (LN) transformation on the initial dataset and then this change was verified further by applying a non-graphical statistical test. It has been observed that initially, the significance value of the test was below 0.05, which shows “the data significantly deviates from a normal distribution”.

Table S4 shows the distribution of original project data (in the left half) and the LN transformation of selected ESCPQC related metrics (in the right half). Both graphical and non-graphical methods were used to assure the LN transformation which has only improved the distribution of project for three metrics. The distribution of 49 metrics out of 51 metrics deviates from normality, which shows a risk that the statistic will be inaccurate. Therefore, the safest remedy is to use an equivalent non-parametric statistic to conclude posted hypotheses.

### Experimental results

The final experimental results could be divided in to two parts:

1. Part A (Derivation of Significant Metrics): This part is derived after first applying Statistical Data Analysis Methods on the collected data that includes checking of relationship significance by applying Correlation between Quality Attributes (the Significant Metrics under ESCPQC classes, which are the metrics or research factors) and the SPI.
2. Part B (Deriving Benefit Reports from Project Data): After identifying significant metrics, which shows good relationship strength with SPI were then used to fetch or generate the final SPI benefit reports directly from the originally collected project data, following the scheme given in Fig. 1.

#### Part A: derivation of significant metrics

##### Hypothesis checking

In order to find the answer to RQ1 the posted hypotheses were checked, the nonparametric replacement of parametric ANOVA test *i.e.*, Kruskal Wallis (KW) along with its pair-wise comparisons was applied. It examines the relationships and differences in between the means and median of three or more independent (unrelated) groups, where SPI was used as a grouping variable. To assess the hypotheses, it uses chi-square for evaluating differences in mean ranks (Green & Salkind, 2013) across the groups. The results of the analysis indicate that there is a significant difference in the medians of 42 metrics out of total 59 metrics with the value of the degree of freedom (df) = 5, the total number of dataset values (*N*) = 62 and significance value (*p*) < 0.05. Therefore, it clearly rejects the posted Null Hypothesesin favor of H1. Furthermore, the “Effect Size” measure was used in the Kruskal-Wallis test, using the chi-square value. Moreover, the strength and direction of the relationship between two quantifiable metrics were calculated using the Spearman correlation coefficient (SCC) (Seltman, 2013). Table S5 displays detailed results for a few sample metrics. In Table S5 column 3, 4 and 5 shows the Spearman correlation test (SCT) at *p* sig value < 0.05 (column 6) to exactly know the strength (strong, moderate, or weak) and direction (direct, inverse, or not a linear relationship) of the relationship between two quantifiable variables. Whereas, column 7 and onwards shows Kruskal Wallis test at sig value < 0.05 (column 6) to know that on changing SDP levels, the median metric values are either significantly different or same.

##### Significant results

To answer RQ2, discussed below are the significant results of Kruskal Wallis test explained by the changing levels of SPI. The SCT results show the increase or decrease in benefits, and the reason of observed behavior in the context of defined rules as in report (Jones & Bonsignour, 2011).

Under the category of product size following are the five basic metrics that will be used further to measure other ESCPQC related metrics. The results indicate that the increasing levels of SPI explain about 28% decrease in the “EquivalentSizeLOC” metric value, a moderate indirect relationship between the two metrics, and an overall increase in benefits. One major reason for this is the achieved stability through code reuse.

Although, increasing levels of SPI does not enforce any constraint of software customization which involves high code reuse this has been observed that software coding practices are also improving as the company is growing mature especially when they are working on Product based development. For “PercentReusedCode” metric the results show that the increasing levels of SPI explain about a 100% increase in the metric value, a strong direct relationship between them, and an overall increase in benefits. This increase in reusability with the increasing maturity levels is due to the general trend of using high reuse of coding practices in product-oriented companies. For “PercOfCodeDiscardedDueToReqVolatilityLOC” metrics the results indicate that the increasing levels of SPI cause about 100% increase in the metric value, indicates a strong direct relationship between the two variables, and an overall reduction in benefits.

With increasing maturity, companies give high priority to client satisfaction. Therefore, change requests increases. And for “PercOfCodeAddedModifiedInMaintPhase” metric the results show a 100% decrease in metric value, a strong indirect relationship between the two metrics, and an overall increase in benefits. When code reusability increases, stability, and quality are observed in delivered applications. The Effort class category includes eleven basic Effort related metrics and Schedule class has four metrics, which will also be used to further measuring other ESCPQC related metrics. In the case of “EffortHour_Actual”, “EffortHour_Planned”, “Schedule_Actual”, and “Schedule_ Planned” the increasing SPI levels shows an increase in the schedule as well as an increase in the effort. Whereas, both “EffortHour_Variance” shows a 36% decrease and “Schedule_Variance” shows a 78% decrease in their values, which shows an increase in benefits as the gap between the actual and predicted values have been reduced, that shows accuracy in estimates.

The answers of RQ2 and acceptance of hypotheses to check the Significant Relationship in between Changing Levels of SPI and ESCPQC metric classes has produced a change in 12 out of 12 metrics related to Effort Class, 4 out of 4 metrics related to schedule class, 4 out of 4 metrics related to cost, 1 out of 1 metric related to productivity class, 18 out of 23 metrics related to quality and 1 out of 1 metrics related to customer satisfaction.

This section discussed the detail of experimental results for only a few sample metrics under each class due to space consumption. In this study, only 29 metrics were taken as suitable for measuring SPI benefits.

##### Insignificant results

Table S5 has also shown insignificant results, which could not be used for measuring SPI benefits in general. The insignificant metrics include “CodeSizeInFPBackFired”, “NumberOf Functional RequirementsDemandedByCustomer”, “NumberOfFunctionalRequirementsDeliveredTo Customer”,“TechnologySuitabilityRequirementCompletionRatio”, “UsabilityOperatorError”, and “UsabilityPercentComfortabilityofOperatorInOperating Computer”. In this section only a few significant benefit results were presented from the actual of 44 out of 55 SPI benefit measurement metric, in “Discussion” covers detailed discussion on the result with the major exceptions in detail.

#### Part B: deriving benefit reports from project data

Next SPI benefit reports are generated from the organization’s collected data, in terms of the significant metrics identified in Part A, it retrieves the quantitative median benefit data from EDA Table S3, and the ESCPQC related auditing reports are then generated in a typical percent benefit increase or decrease, few examples of which are presented in Table S6 that stands over the actual project data, to generate auditor friendly performance reports. In this table the median metric values on specific maturity levels were used to calculate the percent increase or decrease in benefits across the changing levels of SPI. The (+ sign) shows an increase and (– sign) shows decrease across different SPI levels. Through this report, instead of relying over an overall trend, more in-depth trends are evident (See “Deriving Un-Biased Benefit Report” for relevant discussion).

#### Research reliability and validity

A major goal of this study was to develop a reliable and valid conceptual model that exhibited properties of internal reliability and external validity. In this study, the method of exploring new conceptual model is used for exploring higher level relationship between the SPI and the ESCPQC metric classes. According to the defined categories, as recommended in research (Seltman, 2013), the major threats to the validity of this study are as follows.

1. To avoid “Reliability” related biases, only those projects were included in this research which was not presented for actual CMMI appraisals, because there was a high chance of data manipulation to justify the benefits of high maturity practices, especially while apprising high Maturity Levels ML4 and ML5 during appraisals.
2. To avoid “Internal Validity” the key metrics are selected as the ones that have shown high correlation and justify high percent of variability in the metrics using the Kruskal Wallis test (see “Conceptual Model of Research”). For example, on changing levels of SDPI the metrics “PostReleaseDefectsIdentifiedInCode” and “ProductStabilityNumberOfFunctional RequirementChangedAfterSRSSignedOff”, alternate hypothesis is rejected as they do not show direct relationship with SDPI, therefore it is expected that other factors are also impacting the decrease in its value. So, this research is sound and do not include the metrics which show no strong and direct relationship with SDPI.
3. The “Validity of Construct” is checked to assure the constructs or metrics used were relevant measures with the study (see “Methodology”). It assures that the measures correlate with other measures for which it should correlate and should not depend on who is administering the test. Cronbach’s Alpha test was performed to assess the reliability of all ESCPQC constructs (See “Questionnaire Reliability”).
4. For “External Validity” the null hypothesis has been rejected on 0.05 Type I error, that guarantees that there will be only 5% chances of incorrectly rejecting it and that we are 95% confident on reported values of difference in mean outcome. The data collected from a few but the best representatives of Pakistani companies, were sufficient to draw general conclusions. And the selection of candidate metrics could be used as a global contribution in benchmarking SDPI benefit.
5. The “Validity of Conclusion” is about making right statistical conclusion by knowing the degree to which the conclusion we reach is credible. The conclusion is derived from following major issues:

- “Low degree of statistical power” set for Type I error, the experiment performed was a powerful one and statistical analysis has appropriately control Type I error at alpha value 0.5 (see in “Experimental Results” for table on Test of Hypothesis on ESCPQC related Metrics).
- “Fishing and error rate problems” are avoided as the reported results were performed on fixed hypotheses while setting alpha value 0.5, whereas, the tests used are ANOVA equivalent non parametric tests of Kruskal Wallis, which is an exception of alpha inflation problem and is reliable (Trochim & Donnelly, 2006) (see in “Research Question and Hypothesis”).
- To avoid “Low reliability of measures” the measuring instrument has been checked for confirming that there should be no multiple interpretations in metric heading and definitions and also there should be no inappropriate and unclear wordings by authors then evaluated and Pilot tested by two field experts. The field experts evaluated the questionnaire validity and senior Quality Manager from intended domain pretested it (see in “Data Collection and Response Rate”).
- “Low reliability of treatment” is avoided by controlling inconsistent data collection process that normally affects the visibility of relationship between the target and other factors. This study has followed a strict process (see “Data Collection Process” and “Pilot Testing”).
- “Random heterogeneity of respondents” is controlled by collecting data only from selecting homogeneous respondents *i.e.*, from quality management team of higher maturity organizations (see in “Targeted Population and Sampling Method” to “Questionnaire Design”).

## Discussion

Earlier in this report, the reasons for the significant benefit results were presented for a few metrics from a total of 44 out of 55 SPI benefit measurement metric, in this section the major exceptions are being discussed.

### Major exceptions in experimental results

#### Non-significant metrics

The data for the two non-significant metrics *i.e.*, “UsabilityOperatorError” and “PercentUsabilityComfortabilityofOperatorinOperatingComputer” was excluded as it has similar values among all records and is advised to be maintained properly in their historical records’ data repository. To know the importance of these non-significant metrics for SPI benefits after a detailed discussion with Quality Managers the following conclusions were derived:

1. “UsabilityOperatorError” metric is linked with CMMI’s ML3 in Requirement Development Process Area and is identified during User Acceptance Testing activity.
2. “UsabilityPercentComfortabilityOfOperatorInOperatingComputer” metric is not linked with any of the Process Area directly. As CMMI covers Team Training for the development team but not Client's Training.

#### Significant metrics

In the list of 44 significant metrics, 30 metrics have shown a direct relationship between the metric and SPI and shows increasing benefits. Whereas, 14 metrics have shown an indirect relationship between the two thus shows a reduction in benefits include “Schedule_Actual and Schedule_Planned,” “WorkingHoursInMonth,” “PercentageofCodeDiscardedDueToRequirement Volatility,” etc. Similarly the “PercentEffortSavedThroughReuse” increases at High Maturity levels and also the “EffortSpentInRework” decreases. The Quality related metrics such as “PostReleaseDefectsIdentifiedInCode” and “PostReleaseDefectsIdentifiedInDocumentation” increases, because the “NumberofFunctionalRequirementsChangedAfterSRSIsSignedOff” and “ProductStabilityRequirementsVolatility” *i.e.*, “FunctionalRequirementsChangedToDmandRatio” also increases. The high maturity organizations adopt the acceptance of tolerable change request even for non-agile projects to gain client satisfaction and for agile projects this behavior is common. For “PostReleaseDefectsIdentifiedInCode” and “ProductStabilityNumberOfFunctional RequirementChangedAfterSRSSignedOff”, the alternate hypothesis is rejected as they do not show direct relationship with SPI, therefore it is expected that other factors are also impacting the decrease in its value and it is unavoidable to study them with more data in future research.

#### Deriving un-biased benefit report

The quantitative benefit report generation from the actual project data reported in a style normally for which data were collected in qualitative form, is a second major contribution of this research. To avoid biases, an extra care has been taken at the time of project selection, and only those projects were included in this research which were not presented for actual CMMI appraisals, because there was a high chance of data manipulation to justify the benefits of high maturity practices, especially while apprising high maturity levels ML4 and ML5 during appraisals.

The quantitatively calculated median values in Table S6 are calculated from the actual project data. Whereas, the usual practice during appraisals is that, the appraising teams qualitatively collect this data from the organization using process performance report. So, there are high chances of biasness involved in the supplied data or facts by the reporting organization.

Table 3 shows the same overall benefit trend but with level wise depth, such that the “Effort” metric has low value in among the low maturity levels, which then increases till Maturity Level ML3 and finally comes down at high maturity levels of ML4 and ML5.

**Table 3:** Comparison of results with other researches in terms of mean and median values.

Metric	Data points	DACS mean and median (Mcgibbon, Ferens & Vienneau, 2007)	Data points	SEI mean CMMI V 1.0 (Gibson, Goldenson & Kost, 2006)	Data points	SEI mean CMMI V 2.0 (ISACA, 2020)	Data points	This research’s mean and median	Benefits Increased/Decreased
Effort					N/A			Mean: −4% decrease Median: −20% decrease	Increased
Schedule	5	Mean: 32.6% decrease Median: 38% decrease	22	50% (2–95) reduce		95% goals achieved	62	Mean: 51% increase Median: 0% increase	Decreased
Project Cost	2	Mean: 30% decrease Median: 30% decrease	29	34% (3–87) reduce		42% objectives meet	62	Mean: 150% increase Median: 138% increase	Increased
Productivity	12	Mean: 57% increases Median: 39% increase	20	61% (11–329) increase		13–40% increase	62	Mean: 6% increase Median: −0.57% decrease	Increased
Quality	1	Mean: 98% increases Median: 98% increases	34	48% (2–132) increase		25.46% increase	62	Mean: −25% decreased Median: −9 % decreased	Increased
Customer Satisfaction			7	14% (−4 to 55) increase		5–10.3% increase	62	Mean: 9% increase Median: 5% increase	Increased

Whereas, schedule increases up to ML2, then shows a high decrease in value after attaining ML3, but again consumes huge time while attaining ML4 and ML5 level practices. ML4 and ML5 need a high support for automated tools (ISACA, 2020) to perform statistical project management, casual analysis and resolution, organizational process performance and management activities. Because of the lack of understanding, organizations in developing countries do not make right investments. The reason being the majority of work has been done manually which is time-consuming or the low hiring trend of a human resource causes burdening of the available team on multiple tasks. Schedule metrics at ML1, ML2 reflects “The introduction of change effect” which is a general phrase used to reflect an overall phenomenon of an initial loss in performance productivity when an organization adapts major SPI changes until reaching high maturity areas or it gets stable and shows a targeted boost in performance in our study as shown at ML3. Where, lack of understanding and tool support again increases schedule values. This is the point exactly reflected here to build trust in new SPI initiatives (Patric, 2008).

### Comparison of results with other studies and reason of different behavior

In last Table 3 shows a comparison of results among this research and the existing similar researches on measurement and benchmarking attempts for SPI (Gibson, Goldenson & Kost, 2006; Mcgibbon, Ferens & Vienneau, 2007; ISACA, 2020). It has shown that at some points our results are in contrast to the results of DACS, SEI, and ISACA study as we are focused over the early adopters and “the introduction of change effect” (Patric, 2008). Following are the major differences observed:

1. Normally the increasing levels of SPI reflect a decrease in “schedule” value but here the schedule value shows a decrease in benefits. Whereas, ML3 shows optimum point where schedule metric has shown highest benefits.
2. For an increase in “ProjectCost” with the increasing levels of SPI, it could be interpreted as a decrease in benefits from an international perspective that exists in technologically advanced countries (Jones & Bonsignour, 2011).
3. In the other hand, for under developed countries, it is considered as a competitive advantage therefore, it is interpreted as an increase in benefit.

The major reason for the difference in our results’ behavior with the studies conducted in advanced countries may be because the IT industry of outsourcing countries like Pakistan is not very mature both in terms of their origination, use of mature practices and tools at ML4 and ML5.

### Guideline for using research output in real life projects

#### Right mechanism for benefit report generation

Organizations need to enter data following the right mechanism. The Software Engineering Institute follows a higher-level approach for SPI benefit measurement data. It collects percent change (increase or decrease) data readings related to benefit measurement classes using excel sheet-based performance report templates as shown in Fig. S3.

These questionnaire or Performa are recorded as a part of the auditing process on achieving consecutive maturity level. It is compulsory practice for CMMI level 2 and above organizations to collect software metric data. Their project level data set can be examined to generate benefit report. These organizations should follow a bottom-up approach for data collection, according to which it first collect organization’s project level data related to project performance data. While measuring benefit data the organizations with some Nth (0: lower half of Level 1, 1: upper half of level 1, 2, 3, 4, 5) maturity level will share their completed project's benefit data. This then will be used to generate organization wide SPI benefit reports. CMMI ranking wise higher-level improvement benefit information should be generated from the organization’s project level benefit-performance related prediction data stored in project database.

#### Utilization of major outcomes in real projects

As depicted in Fig. 1 the candidate organization can generate the following benefit reports:

1. Actual Quantitative Benefit reports against SPI benefit measurement classes ESCP. These reports are built upon the aggregated median project level prediction data (as generated in Table S6), which is an auditor-friendly version to be utilized in the performance report template in Fig. S3 under the columns “Business Objectives” and “Related Objectives” pointed with arrows.
2. Qualitative benefit reports are generated from above reports, which are generalized percent form, as in Fig. S3 under column “Measurement KPI Target” and “Measurement KPI Actuals”. It is responsibility of an organization to generate it and later link with the appraisal team’s performance report as in Fig. S4B column “Business Benefits”.
3. Same qualitative benefit reports as discussed above are also used by SEI to report executive summary of performance benefits (see Fig. S4B) and it also makes the benefit benchmarks comparable with other benefit studies (see “Comparison of Results with other Studies and Reason of Different Behavior”).

### Research contribution

In reference to the gap analysis (see “Proposed Research Scheme” and “Related Work and Gap Analysis” Table S1) and Research Questions (see “Methodology”), this research has successfully demonstrated following major contributions:

- it quantified the relationship strength between SPI levels and benefit measurement classes namely effort, schedule, cost, productivity, quality, and customer satisfaction (ESCPQC).
- it defined a basic set of significant metrics from different software engineering fields.
- Based upon the significant metrics, it reported cross-organizational SPI benefit benchmarks.

### Applicability and future extensions

The proposed framework, is not dependent any particular technology trend being used in normal SME based studies and emphasizes on reflecting neutral settings. Benchmarking facilitates the outsourcing countries who need low-cost tools for adopting SPI. They do not invest in expensive tools because they are unable to rationalize the associated ROI of these tools (Jones & Bonsignour, 2011). The reason why we could not rely over pure agile based adaptations of SPI, in spite it complements CMMI, because software size is an important factor which if enters in a limit of 1.5 K function points to 100 K function points then formal control points such as change management, design, and code inspections are essential and agile can no longer be fully used in conjunction with CMMI and development must become more formal (Jones, 2008). Another research is conducted on organizations previously certified CMMI V 1.3 more than 10 years back on agile based projects, with just 14% sample representation from the developing country Egypt, concludes that ASD is easily compatible with Level 2 and not completely with Level 3 organizations and that implementation of new rules always require extra time and effort (Ferdinansyah & Purwandari, 2021). Regarding the future extensions of this study and how the proposed framework will adjust the techniques like DevOps, Agile, Cloud and IoT software, data collection is currently focused on Software Development of “Business and Finance” domain software following the iterative waterfall model.

## Conclusion and Recommendations

This research establishes a benchmarking program of SPI benefits for large to medium size software development organization. The data from 62 projects have been used to measure the impact of SPI benefits over ESCPQC related metrics. This research has successfully addressed all major problems and provides answers to the research questions. In this study 44 candidate metrics were identified from an initial set of 50 metrics as determinants of SDPI change in a cross-organization environment. All candidate metrics were classified under six major classes of ESCPQC. It is also demonstrated how to fetch SPI benefit reports for appraisals from a direct project data.

The data analysis has shown improved benefits in 30 metrics under four classes *i.e.*, effort, productivity, quality, and customer satisfaction with the increasing level of SPI, whereas a total of 14 metrics under other two classes *i.e.*, schedule and cost has shown a different behavior.

**Figure 3:**
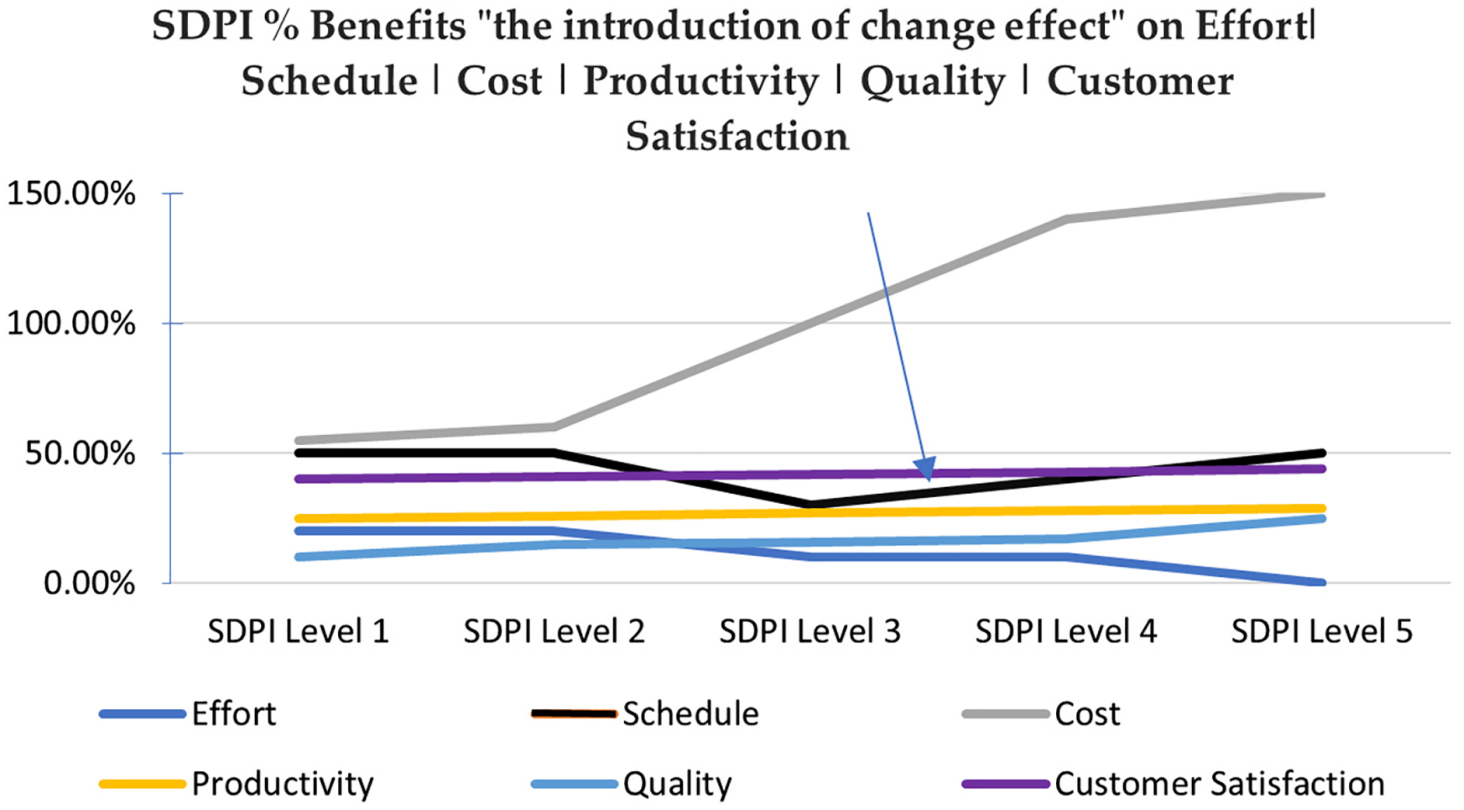
Perceived benefits of this research and the introduction of change effect. There was a reduction in benefits in terms of schedule at maturity level 3 and then a smooth increase at maturity level 4 and 5.

In Pakistan as a developing country more software fee is charged when organizations get more mature to improve their ROI. Therefore, increase in cost is taken as a positive trend. The negative aspect is that this may be the impact of low investments in acquiring quality related expert manpower with good understanding and supporting tools for effective SPI implementation at high maturity levels of ML4 and ML5 that naturally increases Schedule value even after reaching a high reduction at ML3. Auditing practitioners of SEI calls this phenomena as “The introduction of change effect” which shows an initial loss in performance productivity on adaptation of major SPI changes (Patric, 2008) (see Fig. 3). The research reliability and validity is assured by using a reliable instrument and the consistent dataset to build benchmarks. Whereas, generalization is bounded to the business, finance and management information system related application categories from medium to large sized organizations. Hopefully the exact quantification presented in this research will help to build trust in new SPI initiatives. This research application includes the benefit report generation mechanism that would provide insight into Process Performance Baselines (PPB) through ESCPQC benchmarks. This can be used as key performance indicators (KPI) in performance reports. Organizations can use these indicators in managerial-decision-making to avoid cost and schedule over run. This work can be extended in two ways. The data used for analysis is from a developing country, it would be interesting to use data from technologically advanced countries for further assessment. Metrics like prevention cost and return on investment can also be added to study to set an in-depth picture.

## Supplemental Information

10.7717/peerj-cs.936/supp-1 Supplemental Information 1 Research Questionnaire and Collected Data. Each data point in “Raw Data” indicates different metrics data to quantify SDPI benefits

10.7717/peerj-cs.936/supp-2 Supplemental Information 2 Gap areas and their interaction to generate benchmarks.

10.7717/peerj-cs.936/supp-3 Supplemental Information 3 Research Questionnaire Selected Screens.

10.7717/peerj-cs.936/supp-4 Supplemental Information 4 Performance Report Template.

10.7717/peerj-cs.936/supp-5 Supplemental Information 5 SEI reported benefit data. (A) Schedule summary benefits/impacts from multiple organizations and (B) Performance gains

10.7717/peerj-cs.936/supp-6 Supplemental Information 6 CMMI benefits benchmark studies - GAP Analysis.

10.7717/peerj-cs.936/supp-7 Supplemental Information 7 Metric Definitions.

10.7717/peerj-cs.936/supp-8 Supplemental Information 8 EDA Descriptive Statistics on Reported Results.

10.7717/peerj-cs.936/supp-9 Supplemental Information 9 Normality test before and after applying Log (LN) Transformation.

10.7717/peerj-cs.936/supp-10 Supplemental Information 10 Experimentation Detail.

10.7717/peerj-cs.936/supp-11 Supplemental Information 11 Calculation of Quantitative Median and Qualitative Percent Benefit Achieved at different SDPI levels from EDA data.

## Additional Information and Declarations

### Data Availability

The following information was supplied regarding data availability: The raw data is available in the Supplemental File.
