# Supplementary figures and images for: A benchmarking program to support software process improvement adaptation in a developing country, a Pakistan case
Source: PeerJ Comput Sci. 2022 Apr 27;8:e936. doi: 10.7717/peerj-cs.936 (PMC9137942; doi:10.7717/peerj-cs.936)

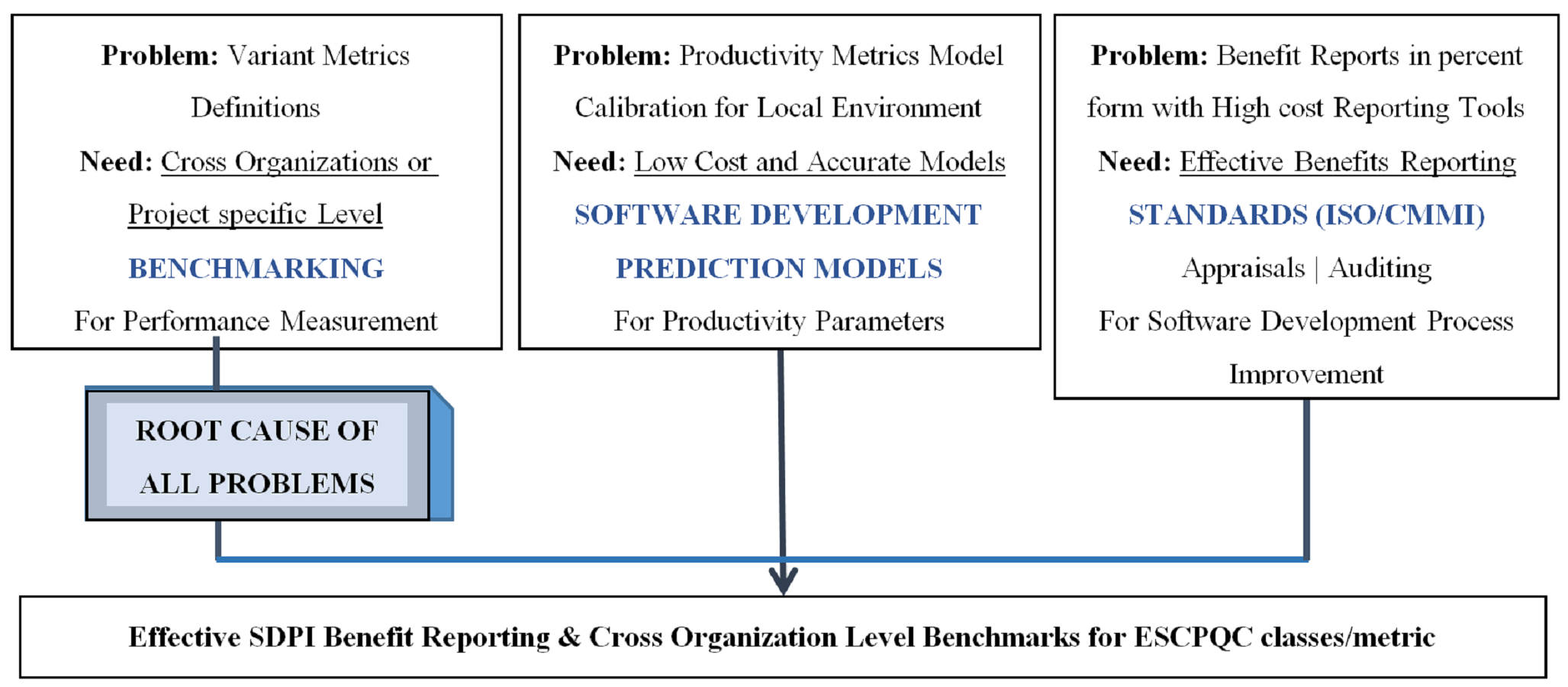

Supplement: Supplemental Information 2 [file peerj-cs-08-936-s002.png]

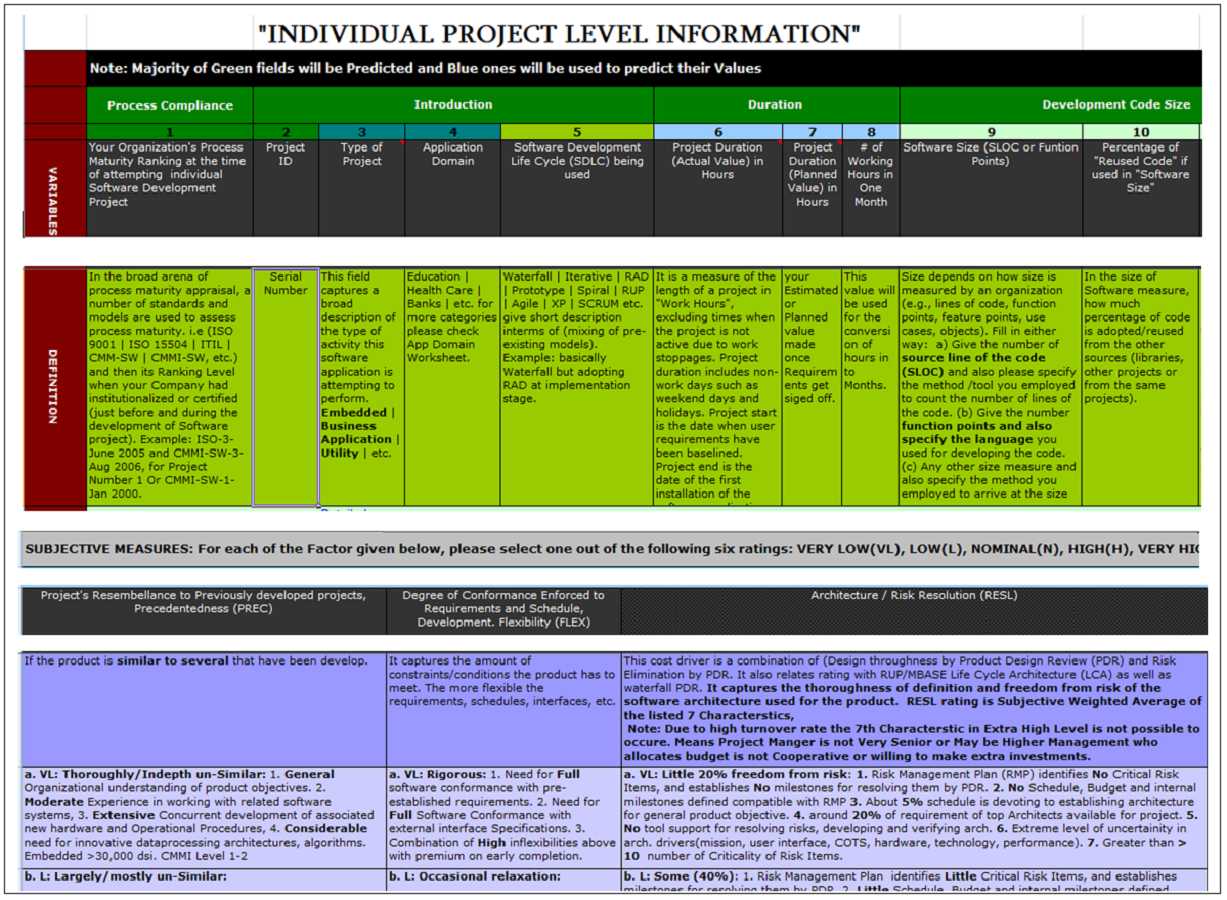

Supplement: Supplemental Information 3 [file peerj-cs-08-936-s003.png]

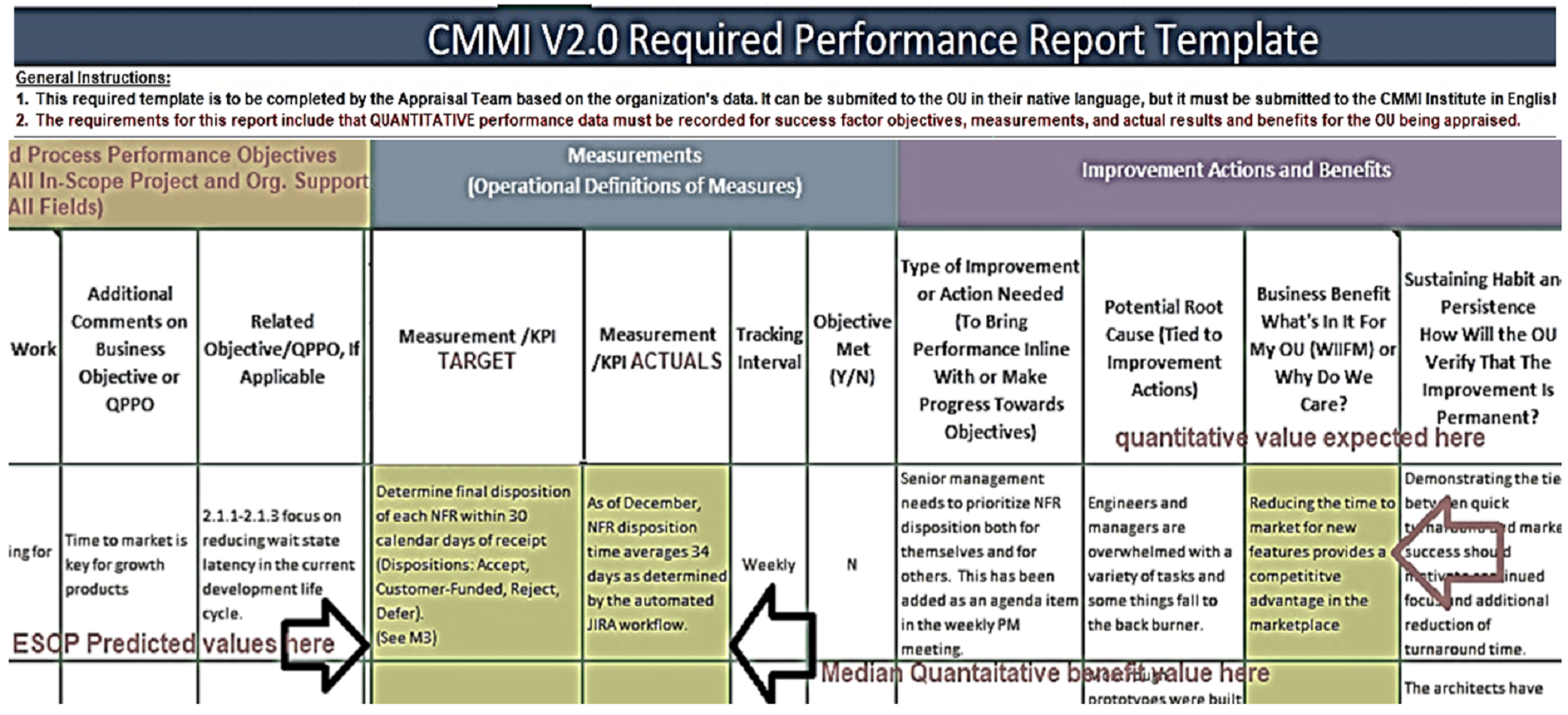

Supplement: Supplemental Information 4 [file peerj-cs-08-936-s004.png]

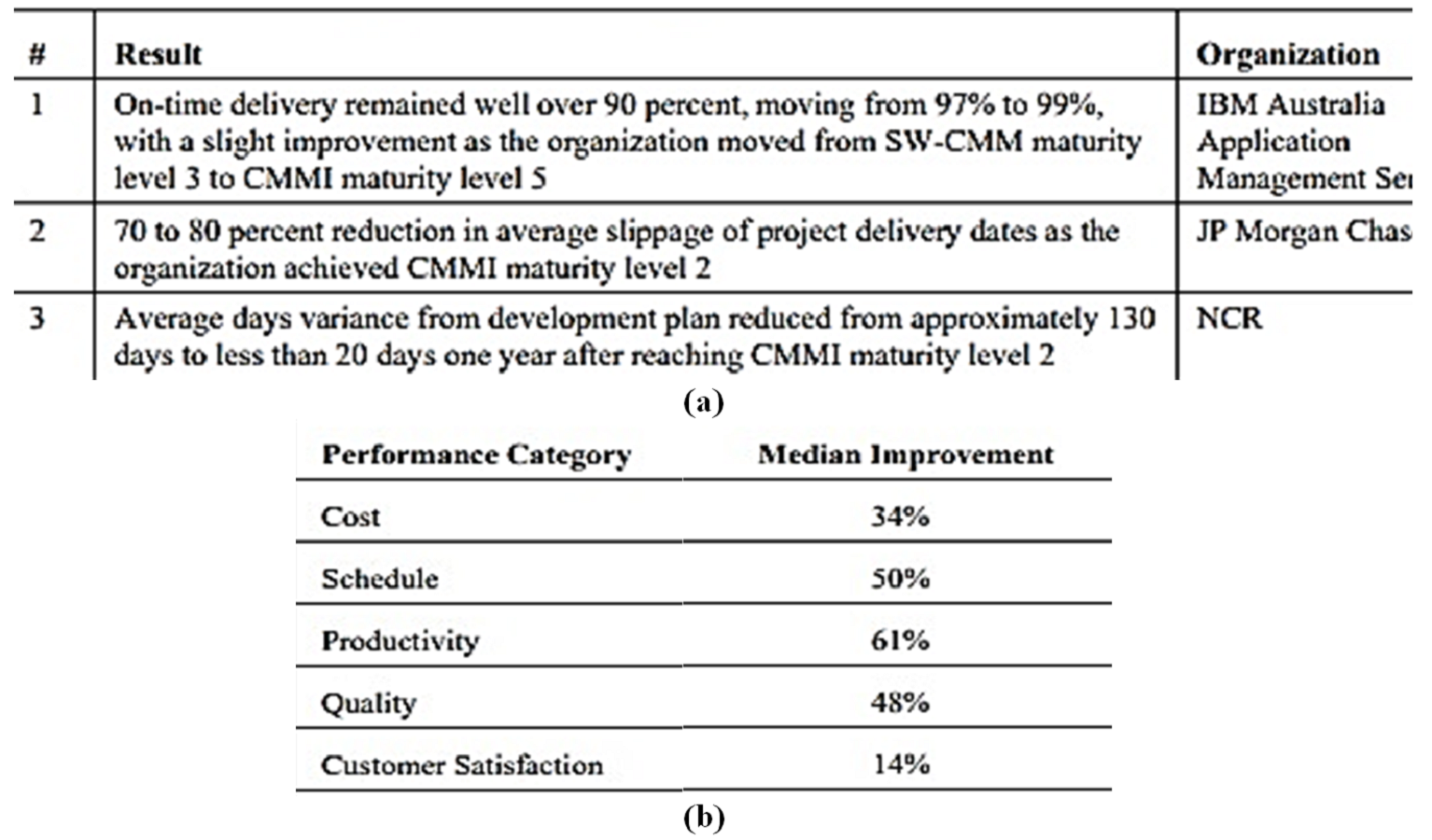

Supplement: Supplemental Information 5 — (A) Schedule summary benefits/impacts from multiple organizations and (B) Performance gains [file peerj-cs-08-936-s005.png]
