# Supplementary material for: A benchmarking program to support software process improvement adaptation in a developing country, a Pakistan case
Source: PeerJ Comput Sci. 2022 Apr 27;8:e936. doi: 10.7717/peerj-cs.936 (PMC9137942; doi:10.7717/peerj-cs.936)
Supplement: Supplemental Information 6 [file peerj-cs-08-936-s006.docx]

| Study | Sub Area | Dependency on Specific External Techniques | Study Region: for Advanced countries (T) or Developing countries (E) | Benchmarking scope and addressment of major three issues | | | | |
| --- | --- | --- | --- | --- | --- | --- | --- | --- |
|  |  |  |  | Statistical Significance of impact | Generalization of Measurements across | | Addressed Problem 1: use Standard metric definitions  cross organization wide | Addresses Problem 2: Benefits reported from project data then represented in Percent Form |
|  |  |  |  |  | Cross Organizations | Single Organization |  |  |
| (Gibson et al., 2006) | Software Development | None | (T) | X | √ | X | X | X |
| (Mcgibbon et al., 2007) | Software Development |  | (T) | X | √ | X | X | X |
| (Goldenson, 2003) | Software Development |  | (T) | X | √ | X | X | X |
| (Al-Yahya et al., 2012) | Software Development |  | (T) | √ | √ | √ | √ | √ |
| (Jones C., 2012) | Software Development |  | (T) | √ | √ | X | √ | X |
| (Singh et al., 2020) | Software Development |  | N/A | √ | √ | √ | √ | X |
| (ISACA, 2020) | Software Development | High dependency on Agility and DevOps | (T) | √ | √ | X | √ | X |
| This Research | Software Development | None | (E) | √ | √ | √ | √ | √ |
|  |  | Note: √ Inclusion and X shows Exclusion of option | | | | | | |
