# Supplementary material for: A benchmarking program to support software process improvement adaptation in a developing country, a Pakistan case
Source: PeerJ Comput Sci. 2022 Apr 27;8:e936. doi: 10.7717/peerj-cs.936 (PMC9137942; doi:10.7717/peerj-cs.936)
Supplement: Supplemental Information 7 [file peerj-cs-08-936-s007.docx]

|  | **Metric with Reference** | **Definition** |
| --- | --- | --- |
|  | SECTION A: Measurement Type and Scale (Objective/ Quantitative) | |
|  | **Project Schedule** (Boehm et al., 2009) |  |
| 1 | Schedule_Actual | It is a measure of actual value of project duration in work hours. Project start is the date when user requirements have been baseline. Project end is the date of the first installation of the software application. |
| 2 | Schedule_Planned | It is an estimated value of project duration in hours. It is calculated once the requirements get signed off. |
| 3 | Schedule_Variance | It is a measure of project delays and is calculated as = (Schedule_Actual - Schedule_Planned) / Schedule_Actual. |
| 4 | WorkingHoursIn Month | A number of working hours in one month. |
|  | **Project Size** (Boehm et al., 2009) |  |
| 5 | EquivalentSizeLOC | Equivalent size of software product measured in Line of Code |
| 6 | CodesizeFPBackFired | Software size in Function Points. |
| 7 | PercentReusedCode | Percentage of "Reused Code" in "Software Size" taken from external sources. |
| 8 | EquivalentReusedCode | It is a measure of product’s equivalent size, which is reused in lines of code (LOC), following COCOMO II standard model definition. |
| 9 | PercOfCodeDiscardedDueToReqVolatility LOC | It is a percentage of code discarded during the project life cycle due to Requirement Volatility. |
| 10 | PercOfCodeAddedModifiedInMaintPhase | In maintenance phase of maximum 6 month duration, percentage of code added and modified. |
|  | **Project Cost** (Boehm et al., 2009) |  |
| 1 | ProductUnitPriceinDollar_Actual (A) | The actual unit price of product in market. Since industry is unable to provide its value and has no standard for setting its value. Therefore, it is calculated as = Effort_Actual * AvgSalaryDrawnByTechPersonnelPerHour. With SPI there is a concept of Cost of Quality (CoQ) advocates for the timewise Effort spent. Whereas, ProductUnitPriceinDollar comprises of Software Development Cost (Cost of Performance) + Cost of Appraisal (Cost of Testing) + Cost of Non-Conformance (Rework). The Cost of Preventions (Process improvement’s plan, review, certification, and training cost) is not included in it and is kept separated and is not the part of this research. |
| 2 | ProductUnitPriceInDollar_Planned (P) | The Planned unit price of product in market and is calculated as = Effort_Planned * AvgSalaryDrawnByTechPersonnelPerHour. |
| 3 | ProductUnitPriceInDollor_Variance | The gap in Actual and Predicted values, and is calculated as = (A - P)/A. |
| 4 | AvgSalaryDrawnByTechPersonnelPerHour | An average salary of a technical staff in dollors. |
|  | **Project Effort** (Boehm et al., 2009) |  |
| 5 | MenPower_Actual (A) | Actual number of technical staff involved in Project. MenPower_Actual is also known as Average Loading. |
| 6 | MenPower_Planned (P) | The Planned value for Menpower. |
| 7 | MenPower_Variance | The gap in Actual and Predicted values, and is calculated as = (A - P)/A. |
| 8 | Effort_Actual (A) | The actual Effort in Hours spent on project-related activities during the life cycle of the project including Requirements engineering, Developer's time spent, Project Management time, Project Support time e.g. (configuration management and quality assurance), and Training time spent over individual project |
| 9 | Effort_Planned (P) | The Planned value for Effort. |
| 10 | Effort_Variance | The gap in Actual and Predicted values, and is calculated as = (A - P)/A. |
| 11 | PercentEffortSavedThroughReuse | From the Effort_Actual, it is the percentage of Effort saved through the Reuse of work products. |
|  | **Effort Distribution** (Boehm et al., 2009) |  |
| 12 | PercentageOfEffortSpentInTraining | From the Effort_Actual, it is the percentage of Effort spent in Training. |
| 13 | PercentageOfEffortSpentInTesting | From the Effort_Actual, it is the percentage of Effort spent in Testing. |
| 14 | PercentageOfEffortSpentInConductingPeer Reviews | From the Effort_Actual, it is the percentage of Effort spent in conducting Peer Reviews. |
| 15 | PercentageOfEffortSpentInTeamMeetings | From the Effort_Actual, it is the percentage of Effort spent in Team Meetings. |
| 16 | PercentEffortSpentInRework | From the Effort_Actual, it is the percentage of Effort spent in Rework, which is usually done for defect removal activities like Review and Inspection. |
|  | **Productivity** (Boehm et al., 2009) |  |
| 17 | Productivity | The work productivity which is a ratio of Output produced in terms LOC with Effort. |
|  | **Quality** |  |
| 18 | PreRelDefectsIdentifiedInSRS | The number of defects identified in System Requirement Specification document (SRS) after SRS is signed off, identified internally. |
| 19 | PreRelDefectsRemovedFromIdentifiedInSRS | It shows the number of defects removed from the identified ones in SRS. |
| 20 | PreRelDefectsIdentifiedInCode | The number of defects identified inhouse in code. |
| 21 | PreRelDefectsRemovedFromIdentifiedInCode | The number of defects removed from the identified ones in code. |
| 22 | PreRelDefectsIdentifiedInArchitectureAndDesign | The number of defects identified inhouse in design and architecture. |
| 23 | PreRelDefectsRemovedFromIdentifiedInArchDesig | The number of defects removed from the identified ones in architecture and design. |
| 24 | DefectDensity_PreRelDefectToSizeRatio | The ratio of total product defects in its code with code size. |
| 25 | PostRelDefectsIdentifiedInCode | The number of defects reported by customers. |
| 26 | PostRelDefectsIdentifiedInDocumentation | The number of Post-Release Defects in Documentation Identified by Customer. |
| 27 | DefectDetectionPercentage_PreReltoPostRelRatio | The ratio of (Pre-Release Code Defects found during QA,Testing stage) with (Pre+Post release defects found by customer after release) |
| 28 | DefectRemovalEffectiveness_PreRelDefFoundinCodeSRSArchPerFromPreandPost | The ratio between (Prerelease defects Found in Code + SRS + Architecture * 100) and (defects Found in Code + SRS + Architecture + Postrelease defects Found in Code + SRS + Architecture) |
| 29 | Defect Removal Efficiency _PreRelDefResolvedRatioPreRelDefFound | The ratio between (Prerelease defects Resolved) and (Prerelease defects Found) |
| 30 | Number of Review changes in Project Plan | The Revisions in Project Plan document throughout the project lifecycle. |
| 31 | Number of Functional Requirements Demanded by Customer | The number of Functional Requirements demanded by the customer, signed off in SRS. |
| 32 | Number of Functional Requirements Delivered to Customer | The number of Functional Requirements delivered to customer out of the total signed off requirements in SRS. |
| 33 | Technology Suitability Requirement Completion Ratio | The measure of Product compliance, and is a ratio between Number of Functional Requirements Demanded and Number of Functional Requirements Delivered. |
|  | **Product Stability** |  |
| 34 | Number Of Functional Reqs Changed After SRS Sign off | The Number of Functional Requirements Added or Deleted after once SRS get signed off. |
| 35 | ReqVolatilityFuncReqChangedToDemandedRatio | Ratio of Total Requirements Changed and Actual Number of Requirements. |
|  | **Customer Satisfaction** |  |
| 36 | PercentageOfCustomerSupportQueriesResolved | The Percentage of Customer Support Queries resolved without extra charges. |
|  | **Usability** |  |
| 37 | Operator Error | Errors produced by the client while using the developed product. |
| 39 | Percent Comfortability of operator in Operating Computer | Rated value of percent comfortability in developed product in between 0% to 100%. |
| 40 | Reliability MTBF | Mean Time To Failure + Mean Time To Restore. |
| 41 | Risks Evaluated During Project Life Cycle | Frequency of potential risk evaluated during project lifecycle. |
| 42 | SECTION B: Measurement Type and Scale (Subjective/ Qualitative) | |
|  | **SDPI or PMAT** (Boehm et al., 2009) |  |
| 43 | Process Maturity Ranking | It is represented as PMAT in COCOMO model manuals and SDPI in this study. Detailed definition of which could be seen in Table 4. |
|  | COCOMO II Questionnaire, Cost Drivers | It comprises of 22 factors under the categories of personnel, product, platform, and project etc. For details check the reference. |
