# Supplementary material for: A benchmarking program to support software process improvement adaptation in a developing country, a Pakistan case
Source: PeerJ Comput Sci. 2022 Apr 27;8:e936. doi: 10.7717/peerj-cs.936 (PMC9137942; doi:10.7717/peerj-cs.936)
Supplement: Supplemental Information 8 [file peerj-cs-08-936-s008.docx]

| Metrics | Changing the level of PMAT Rating | N | Mean | Std. Dev. | Min | Max |
| --- | --- | --- | --- | --- | --- | --- |
| PreRelDefect  IdentifiedInCode | 0 | 17 | 38.05 | 11.13 | 0.00 | 106 |
|  | 1 | 14 | 33.57 | 10.66 | 0.00 | 104 |
|  | 2 | 6 | 14.50 | 4.88 | 0.00 | 42 |
|  | 3 | 4 | 2.00 | 0.00 | 0.00 | 6 |
|  | 4 | 4 | 4.00 | 1.00 | 0.00 | 8 |
|  | 5 | 17 | 8.00 | 2.00 | 0.00 | 10 |
