# Supplementary material for: A benchmarking program to support software process improvement adaptation in a developing country, a Pakistan case
Source: PeerJ Comput Sci. 2022 Apr 27;8:e936. doi: 10.7717/peerj-cs.936 (PMC9137942; doi:10.7717/peerj-cs.936)
Supplement: Supplemental Information 9 [file peerj-cs-08-936-s009.docx]

| Non LN data | | LN data | |
| --- | --- | --- | --- |
| Shapiro-Wilk (df=62) | Sig. | Shapiro-Wilk (df=62) | Sig. |
| Schedule_Actual | 0.000 | LN1_Schedule_Actual | 0.000 |
| Schedule_Planned | 0.000 | LN1_Schedule_Planned | 0.000 |
| Schedule_Variance | 0.000 | LN7_Schedule_Variance | 0.000 |
| EquivalentSizeLOC | 0.000 | LN1_EquivalentSizeLOC | 0.088 |
