# Supplementary material for: A benchmarking program to support software process improvement adaptation in a developing country, a Pakistan case
Source: PeerJ Comput Sci. 2022 Apr 27;8:e936. doi: 10.7717/peerj-cs.936 (PMC9137942; doi:10.7717/peerj-cs.936)
Supplement: Supplemental Information 10 [file peerj-cs-08-936-s010.docx]

| (ESCPQC) Metrics  (# of Significant metrics / Total # of Metrics in this category) | Benefit Metrics | Spearman Correlation Test | | | | | Kruskal Wallis Test | | | | |
| --- | --- | --- | --- | --- | --- | --- | --- | --- | --- | --- | --- |
|  |  | Rs^2^  N = 62 | Derived Conclusion | | Rs  Sig. (p) <= .05 | H1 | Sig. (p)  p < 0.05 | H0 | Effect Size | Chi2  df = 5  N = 62 | Changing SDPI levels explain % Variability in metric value |
|  |  |  | (Increase or Reduction) in benefits | Relationship Strength  (Week <=2 \| Moderate > 2 and < 6 \| Strong > 6), Direction (+ Direct \| - Inverse) |  |  |  |  |  |  |  |
| Schedule  (4/4) | Schedule_Actual | 0.25 | Reduction | Moderate, Direct | .05 | Accept | 0.04 | Reject | 0.19 | 11.66 | 19 % |
|  | Schedule_Planned | 0.67 | Reduction | Moderate, Direct | .00 | Accept | 0.00 | Reject | 0.57 | 34.90 | 57 % |
|  | Schedule_Variance | -0.76 | Increase | Strong, Inverse | .00 | Accept | 0.00 | Reject | 0.78 | 47.57 | 78 % |
|  | WorkingHoursInMonth | 0.87 | Reduction | Strong, Direct | .00 | Accept | 0.00 | Reject | 1.0 | 61.00 | 100 % |
| Size  (4/5) | EquivalentSizeLOC | -0.49 | Increase | Moderate, Inverse | .00 | Accept | 0.00 | Reject | 0.29 | 17.60 | 29 % |
|  | CodeSizeFPBackFired |  |  |  |  |  | 0.13 | Accept |  |  |  |
|  | PercentReusedCode | 1.0 | Increase | Strong, Direct | .00 | Accept | 0.00 | Reject | 1.0 | 60.99 | 100 % |
|  | PercOfCodeDiscardedDueToReqVolatility LOC | 0.91 | Reduction | Strong, Direct | .00 | Accept | 0.00 | Reject | 1.0 | 61.00 | 100 % |
|  | PercOfCodeAddedModifiedInMaintPhase | -0.90 | Increase | Strong, Inverse | .00 | Accept | 0.00 | Reject | 1.0 | 60.99 | 100 % |
| Cost  (4/4) | ProductUnitPriceinDollar_Actual | 0.77 | Increase | Strong, Direct | .00 | Accept | 0.00 | Reject | 0.73 | 44.70 | 73 % |
|  | ProductUnitPriceInDollar_Planned | 0.65 | Increase | Strong, Direct | .00 | Accept | 0.00 | Reject | 0.64 | 38.90 | 64 % |
|  | ProductUnitPriceInDollor_Variance | 0.63 | Reduction | Strong, Direct | .00 | Accept | 0.00 | Reject | 0.43 | 26.37 | 43 % |
|  | AvgSalaryDrawnByTechPersonnelPerHour | 1.00 | Increase | Strong, Direct | .00 | Accept | 0.00 | Reject | 1.0 | 61.00 | 100 % |
| Effort  (12/12) | ManPowerPermonth_Actual | -0.70 | Increase | Strong, Inverse | .00 | Accept | 0.00 | Reject | 0.68 | 41.30 | 68% |
|  | MenPowerPerMonth_Planned | -0.72 | Increase | Strong, Inverse | .00 | Accept | 0.00 | Reject | 0.60 | 36.50 | 60% |
|  | MenPowerPerMonth_Variance | 0.65 | Reduction | Strong, Direct | .00 | Accept | 0.00 | Reject | 0.49 | 29.92 | 49 % |
|  | EffortinHour_Actual | -0.704 | Increase | Strong, Inverse | .00 | Accept | 0.00 | Reject | 0.51 | 36.10 | 51 % |
|  | EffortinHour_Planned | -0.707 | Increase | Moderate, Inverse | .00 | Accept | 0.00 | Reject | 0.33 | 34.21 | 33 % |
|  | EffortinHour_Variance | -0.22 | Increase | Weak, Inverse | .00 | Accept | 0.00 | Reject | 0.31 | 19.10 | 31% |
|  | PercentEffortSavedThroughReuse | 0.97 | Increase | Strong, Direct | .00 | Accept | 0.00 | Reject | 0.95 | 57.82 | 95 % |
|  | PercentEffortSpentOnTraining | -0.62 | Increase | Strong, Inverse | .00 | Accept | 0.00 | Reject | 0.83 | 50.39 | 83 % |
|  | PercentEffortSpentOnTesting | -0.85 | Increase | Strong, Inverse | .00 | Accept | 0.00 | Reject | 0.93 | 56.60 | 93 % |
|  | PercentEffortspentInConductingPeerReview | -0.67 | Reduction | Strong, Inverse | .00 | Accept | 0.00 | Reject | 0.69 | 41.96 | 69 % |
|  | PercentEffortSpentInTeamMeetings | -0.35 | Increase | Moderate, Inverse | .00 | Accept | 0.01 | Reject | 0.26 | 15.60 | 26 % |
|  | PercentEffortSpentInRework | -0.84 | Increase | Strong, Inverse | .00 | Accept | 0.00 | Reject | 0.79 | 48.50 | 79 % |
| Quality  (18/23) | PostRelDefectsIdentifiedInCode | 0.14 | Reduction | Not a linear relationship | .27 | Reject | 0.01 | Reject | 0.27 | 16.43 | 27 % |
|  | ProductStabilityNumberOfFunctionalReqsChangedAfterSRSSignoff | 0.59 | Reduction | Not a linear relationship | .65 | Reject | 0.04 | Reject | 0.2 | 11.97 | 2 % |
| Productivity  (1/1) | Productivity | 0.41 | Increase | Moderate, Direct | .00 | Accept | 0.00 | Reject | 0.61 | 37.02 | 61 % |
