# Supplementary material for: A benchmarking program to support software process improvement adaptation in a developing country, a Pakistan case
Source: PeerJ Comput Sci. 2022 Apr 27;8:e936. doi: 10.7717/peerj-cs.936 (PMC9137942; doi:10.7717/peerj-cs.936)
Supplement: Supplemental Information 11 [file peerj-cs-08-936-s011.docx]

| SDPI Classes | PMAT Rating | N dataset | Mean value of N dataset | % Value increase or Decrease across Different SDPI level |
| --- | --- | --- | --- | --- |
| Schedule_ Actual | 0 | 10 | 1479 | 0 Level of SDPI |
|  | 1 | 11 | 1482 | 0% increase |
|  | 2 | 7 | 1500 | 0% increase |
|  | 3 | 9 | 975 | -0.65% decrease |
|  | 4 | 8 | 1500 | 1.54% increase |
|  | 5 | 17 | 2536 | 1.69% increase |
|  |  |  | Mean: | 0.516 % increase |
|  |  |  | Median: | 0.00% increase |
| Effort_Actual | 0 | 10 | 993 | 0 Level of SDPI |
|  | 1 | 11 | 478 | -0.52% decrease |
|  | 2 | 7 | 854.8 | 0.79% increase |
|  | 3 | 9 | 1433.9 | 0.68% decrease |
|  | 4 | 8 | 2.7 | -0.99% decrease |
|  | 5 | 17 | 2.16 | -0.2% increase |
|  |  |  | Mean: | -0.2% decrease |
|  |  |  | Median: | 0.30% increase |
